# Lack of standardisation in the management of complex tibial plateau fractures: a multicentre experience

**DOI:** 10.1007/s00068-024-02616-6

**Published:** 2024-08-02

**Authors:** Christina Hörmandinger, David Bitschi, Daniel P. Berthold, Claas Neidlein, Lennart Schroeder, Julius Watrinet, Robert Pätzold, Wolfgang Böcker, Boris Michael Holzapfel, Julian Fürmetz, Markus Bormann

**Affiliations:** 1 Department of Orthopaedics and Trauma Surgery, Musculoskeletal University Center Munich (MUM), University Hospital, LMU Munich, Marchioninistraße 15, 81377 Munich, Germany; 2 Department of Trauma Surgery, Trauma Center Murnau, Murnau am Staffelsee, Germany

**Keywords:** Tibial plateau fracture (TPF), Fracture register, Treatment standard, Fracture classification, Perioperative imaging, Osteosynthesis

## Abstract

**Objective:**

In recent years, the trauma mechanisms and fracture types in tibial plateau fractures (TPF) have changed. At the same time, treatment strategies have expanded with the establishment of new classification systems, extension of diagnostics and surgical strategies. Evidence-based recommendations for treatment strategies are rare. The aim of this study is to assess the extent of standardization in the treatment of complex TPF.

**Material and methods:**

For the study, specialists in trauma surgery/orthopaedics were presented thin-slice CT data sets of three complex TPFs including 3D reconstructions. A standardized questionnaire on fracture morphology and planned treatment strategy was then completed.

**Results:**

A total of 23 surgeons from 7 hospitals (Trauma center levels I–III) were included.

All three fractures were most frequently classified as Schatzker type V (fracture I: 52.2%, II: 56.5%, III: 60%). Averaged over all three fractures, 55% of the respondents chose the same patient positioning. The combination of a posteromedial and anterolateral approach was the most frequently chosen approach at 42.7%. Double plating was favored for the surgical treatment of all fractures (70.7%). Preoperative MRI, extended approaches and intraoperative fraturoscopy were significantly more common in level I trauma centres.

**Conclusion:**

There are major differences in the management of complex TPF. 360° treatment is carried out in all departments regardless of the level of care, but without further standardization in terms of preoperative imaging, classification, initial treatment, approach, fixation and intraoperative imaging. There are major differences within the departments with different level of care.

**Supplementary Information:**

The online version contains supplementary material available at 10.1007/s00068-024-02616-6.

## Introduction

Tibial plateau fractures (TPF) pose a significant challenge in trauma and orthopedic surgery, primarily due to their complexity. Currently, the preferred diagnostic tool is computed tomography (CT) imaging, recognized as the gold standard [1], while modern techniques like 3D printing and Mixed-Reality (MR) visualization enhance improved comprehension of the fracture [2, 3]. Presently, there is no generally recommended approach for identifying additional soft tissue damage by Magnetic Resonance Imaging (MRI) in complex TPF cases [1]. This holds clinical significance given the rising incidence of TPF in the past decade [5].

In an ageing society the accident mechanism is shifting towards low-energy trauma with an accompanying change in fracture morphology [1, 4–6]. Conventional two-dimensional fracture classifications, such as those by Schatzker, Arbeitsgemeinschaft für Osteosynthesefragen (AO), and Moore [7–9], are progressively giving way to three-dimensional assessments like the ten-segment classification or the three-column model [10–13].

This shift in fracture analysis has significantly impacted the treatment strategies of TPF. New approaches and step-by-step extension of the existing approaches for better visualisation of the articular surface and treatment of posterior fracture fragments have been developed [4, 14–16]. Despite significant advances in diagnostics and treatment, the incidence of secondary osteoarthritis after TPF remains high, ranging from 13 to 83% [17–19].

In the realm of daily clinical practice, surgeons encounter a diverse range of diagnostic and therapeutic possibilities but evidence-based recommendations are rare [1, 20]. Furthermore, significant disparities in the reliability of different classification systems pose a challenge, raising doubts about the comparability of treatment recommendations and outcome data across studies [3, 21, 22].

Therefore, the purpose of this study was to examine the care strategies utilized for managing complex TPF across different healthcare facilities. We hypothesized that there is no consensus within a cohort of highly experienced surgeons and there would be the need for a more standardized process for the successful treatment of complex TPF.

## Methods

### Case selection

**Fig. 1:**
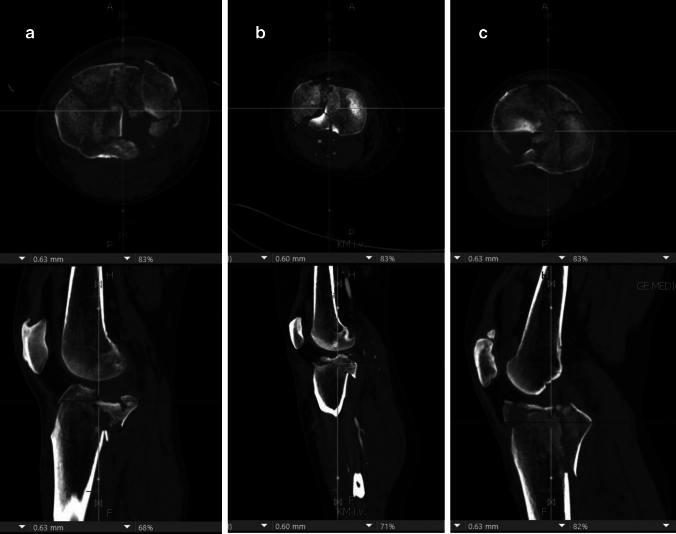
CT (axial and sagittal, coronal not shown) of the three cases. **a** case 1, **b** case 2, **c** case 3

**Fig. 2:**
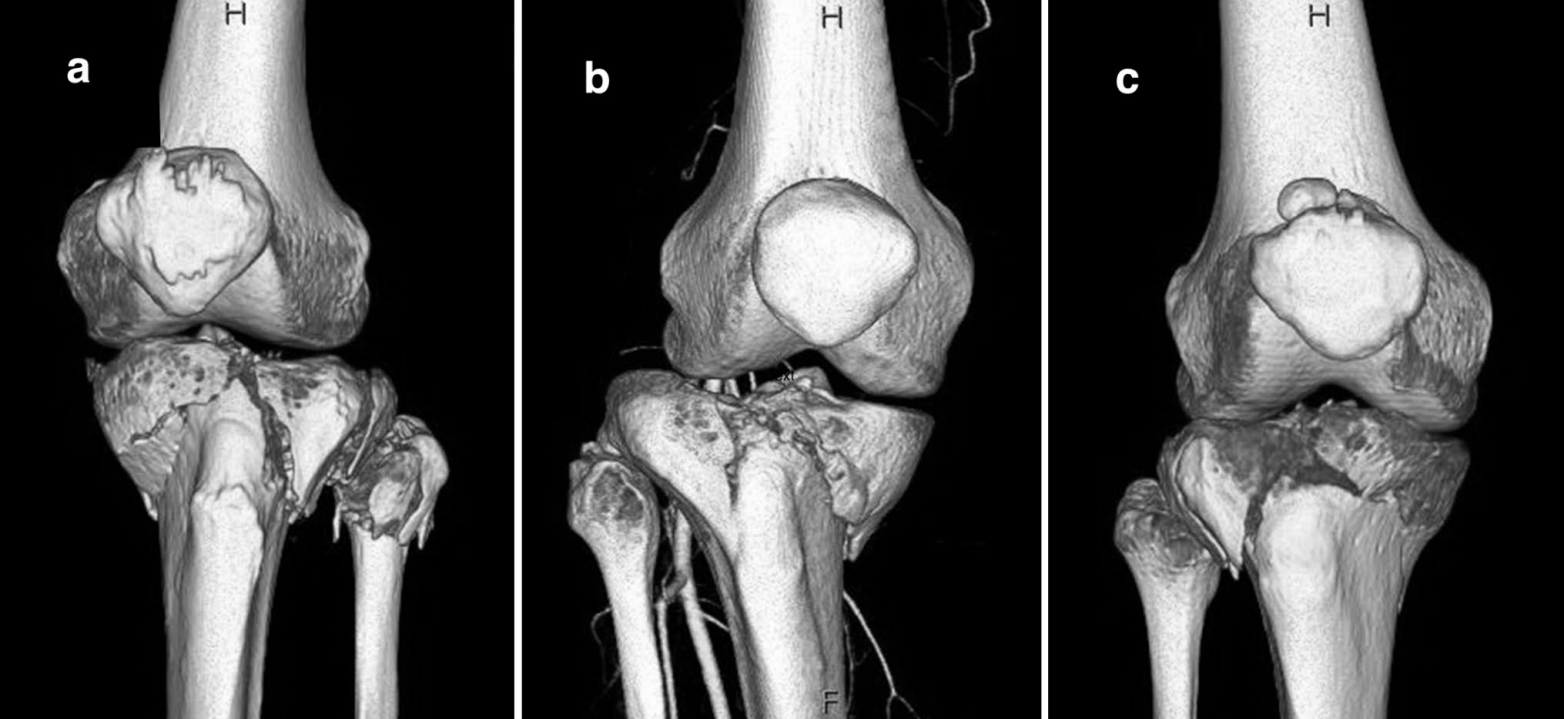
3D CT reconstruction of the three cases **a** case 1, **b** case 2, **c** case 3

Three randomly selected complex TPF from a German level I trauma center TPF database were selected for this study. A 3-Dimensional (3D) CT reconstruction was generated from the available CT thin slice dataset (slice thickness < 0.7 mm). The fractures included bicondylar, anterior, and posterior fracture fragments. CT imaging, along with 3D CT reconstruction, was presented to specialists in trauma surgery and/or orthopedics using Visage 7.1.16 software (Visage Imaging, CA, USA), as shown in Figs. 1 and 2. These specialists, attending specialists in trauma surgery and orthopedics, provided expert insights.

### Specialist selection

A personal invitation was sent to specialists in trauma surgery who work at certified german trauma centers. The cases were presented by one of the study authors (M.B.) to the participating surgeons at the participating hospital. Participating surgeons were also asked if they had intraoperative 3D CT imaging and arthroscopy/fracturoscopy available for treatment.

### Outcomes

A standardized questionnaire on fracture morphology, classification and recommended treatment strategy was completed by each specialist following the case presentation. This questionnaire (Appendix) was created using the web application SoSci Survey (SoSci Survey GmbH, Munich, Germany). All surveyed surgeons had to assess two fractures. Afterwards they were given the choice of taking time to assess an additional third fracture.

### Statistical analysis

The statistical analysis was performed using SPSS Statistics 26.0 software (IBM Corp., Armonk, USA). The statistical test procedures used were the chi-square test and the exact test according to Fischer, with a significance level of p < 0.05. The reliability analysis was used for Fleiss’ Kappa. The graphical representation was carried out using Microsoft Excel 365 MSO Version 2207 (Microsoft Corp., Redmond, USA).

## Results

### Specialist selection

A total of 23 specialist surgeons from 7 different hospitals with varying trauma levels were included. The distribution of the departments' levels of care and the previous number of surgically treated TPF by the respondents is delineated in Table 1. Trauma center level I represents the highest level. Of the respondents, 69.6% (n = 16) had treated at least 50 TPF in their previous professional experience.

**Table 1:** Collective of interviewed surgeons

	Trauma center level I: n = 3	Trauma center levels II + III: n = 4	Total collective: n = 7
Surgeons (n)	10 (43%)	13 (57%)	23
TPF treated surgically (n)			
> 100 fractures	2 (20%)	2 (15%)	4 (17.4%)
51–100 fractures	4 (40%)	8 (62%)	12 (52.2%)
10–50 fractures	4 (40%)	3 (23%)	7 (30.4%)

*TPF* tibial plateau fractures

All surgeons reported that intraoperative arthroscopy/fracturoscopy was available in their department. Intraoperative 3D CT imaging was available at all participating level I hospitals. Two level II/III departments did not have the option of intraoperative 3D CT imaging. In total, 20 out of 23 surgeons had the possibility of intraoperative 3D CT imaging.

Thirteen surgeons assessed 2 fractures, while 10 surgeons assessed 3 fractures. Overall, this resulted in 56 cases/assessments. Table 4 in the appendix shows the most frequently selected answer option for each category.

### Fracture classification

**Fig. 3:**
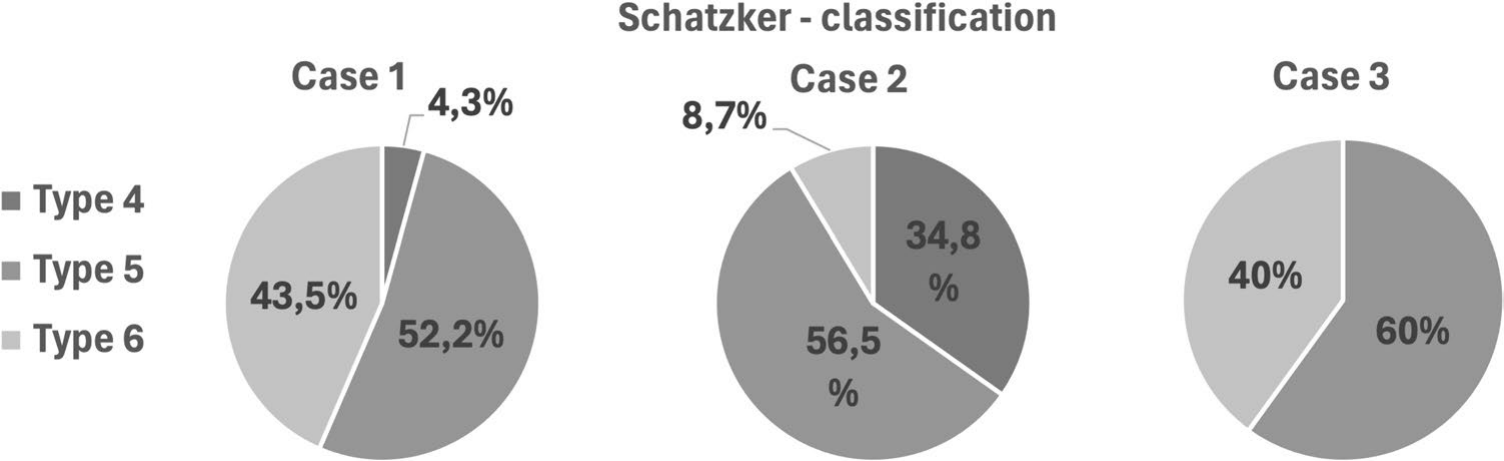
Schatzker classification—distribution of the selected classification based on the cases

When using the Schatzker classification, all 3 fracture cases were most frequently classified as Schatzker 5, accounting for 55.4% (n = 31) of cases, on average. The highest agreement in classification was found for case 3, with 60% (Fig. 3). Table 2 represents the interrater reliability for the Schatzker classification with Fleiss’ Kappa going from 0.620 for the second case to 0.643 for the third case.

**Table 2:** Interrater agreement for Schatzker classification

	Interrater agreement
Fracture 1	0.629
Fracture 2	0.620
Fracture 3	0.643

**Fig. 4:**
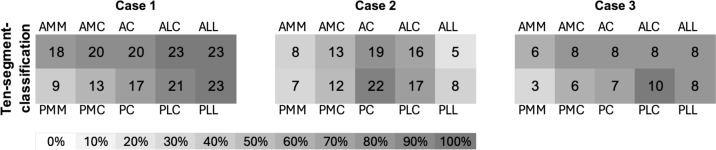
Ten-segment classification—frequency of the selected segments

When using the ten-segment classification, Fig. 4 shows the frequency distribution of the selected segments within the ten-segment classification. On average, 8.1 affected segments were chosen for fracture I, 5.5 for fracture II, and 7.2 for fracture III. The highest agreement in the number of selected segments was observed for fracture 3, with 8 segments at 50% (n = 5). On average, respondents selected an identical combination of segments in 16.1% (n = 9) of all cases. The greatest agreement was found for fracture I, at 21.7% (n = 5).

The patient’s age in the first case was 56 years, in the second case 30 years and in the third case 53 years.

### Preoperative imaging

In addition to CT, respondents expressed a desire for additional MRI in 42.9% (n = 24) on average. The highest agreement was observed for case 2, with 56.5% (n = 13).

### Surgical treatment

The primary treatment for all fractures was most frequently recommended using external fixation, averaging 76.8% (n = 43). Alternatively, initial treatment was recommended using a brace or cast.

None of the respondents opted for Total Knee Arthroplasty (TKA), all favoured osteosynthesis.

Most of the surgeons opted for intraoperative patient re-positioning (prone to supine) for the treatment of case I and III (case I: 56.5% (n = 13), case III: 70% (n = 7)). In contrast, 60.9% (n = 14) of the respondents were planning the case II without re-positioning.

**Fig. 5:**
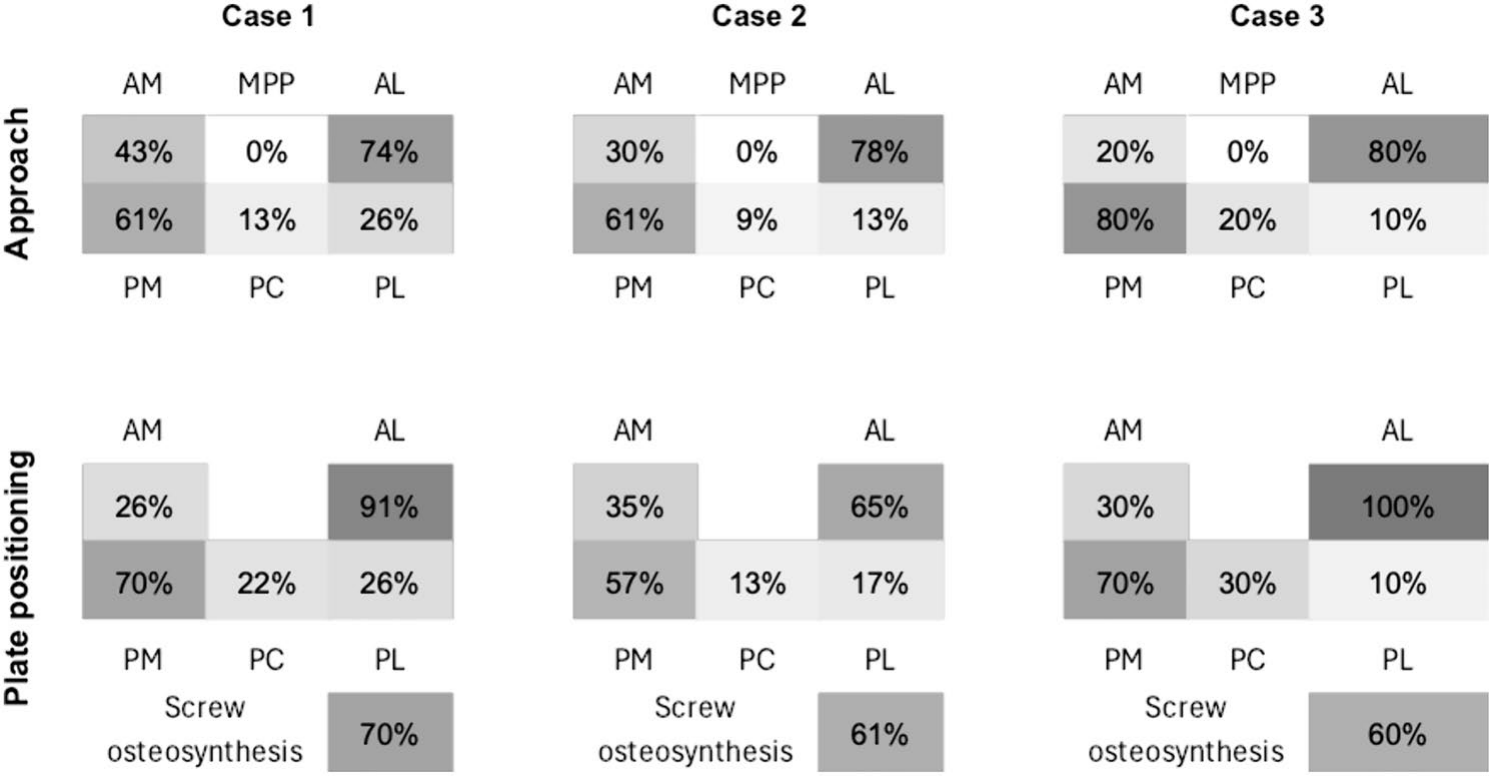
Frequency of approach and plate positioning. *AM* anteromedial, *AL* anterolateral, *PC* posterocentral, *PM* posteromedial, *PL* posterolateral, *MPP* medial parapatellar

Figure 5 illustrates the selected approaches and fixation techniques. In 75% (n = 42) of cases, fracture treatment is planned via combined approaches. The most common combination is an anterolateral and posteromedial approach, accounting for 41.1% (n = 23). Double plate osteosyntheses were most frequently planned (73.2% (n = 41)). Bone augmentation is planned in 78.6% (n = 44) on average, most frequently with allogenic material (60.7% (n = 34)).

Intraoperative imaging is more often done by image intensifier, less frequently with arthroscopy/fracturoscopy.

Table 3 below provides an overview of the differences in treatment strategy between Trauma centers level I and other levels.

**Table 3:** Differences in treatment strategy dependent on level of care

	Trauma center level I (n = 3)	Trauma center levels II + III (n = 4)	p-value
Schatzker type 5	45.8% (n = 11)	62.5% (n = 20)	
Additional MRI	75% (n = 18) yes	18.8% (n = 6) yes	< 0.001
Primary care	83.3% (n = 20) external fixator	71.9% (n = 23) external fixator	0.357
Positioning			
Single positioning	20.8% (n = 5)	65.6% (n = 21)	< 0.001
Intraoperative repositioning	79.2% (n = 19)	34.4% (n = 11)	
Approach			
Multiple approach	87.5% (n = 21)	96.9% (n = 31)	0.303
Including posterior approach	87.5% (n = 21)	81.3% (n = 26)	0.717
Extended approach	37.5% (n = 9)	9.4% (n = 3)	0.019
Osteosynthesis			
Single plate	4.2% (n = 1)	6.3% (n = 2)	
Double plate	75% (n = 18)	71.9% (n = 23)	
Triple plate	20.8% (n = 5)	21.9% (n = 7)	
Additional screw	70.8% (n = 17)	59.4% (n = 19)	
Bone augmentation			
Allogeneic	83.3% (n = 20)	43.8% (n = 14)	0.005
Autologous	0% (n = 0)	21.9% (n = 7)	
Intraoperative imaging			
With 3D	62.5% (n = 15)	37.5% (n = 12)	0.104
With fracturoscopy	37.5% (n = 9)	9.4% (n = 3)	0.019

Surgeons in a maximum care setting (level I) are significantly (p < 0.001) more likely to request additional preoperative MRI. In these hospitals intraoperative repositioning and extended approaches are significantly more frequent (p < 0.001 respectively p = 0.019). Further, the number of fracturoscopies is also significantly (p = 0.019) higher in these hospitals.

## Discussion

The most important finding of this study was that there were significant differences in the management of TPF among highly experienced surgeons, with these differences partly attributed to the level of care of the trauma center. However, whether the different methods and strategies lead to different outcomes and whether this justifies treatment of complex fractures exclusively in maximum care centres has not yet been clarified and remains the subject of further research.

This study calls for further research on different treatment strategies to establish a more standardized approach in the management of TPF to improve outcomes, reduce future complications, and lower conversion rates to TKA.

In the beginning, the classification system should exhibit high validity and reliability, facilitate the derivation of a treatment algorithm, and encompass expected patient outcomes [23]. Miler et al. highlighted the existence of 38 different classification systems for TPF in the literature, with only a few meeting these criteria [22]. Even within the less complex Schatzker et al. classification system, the data from this study demonstrates a maximum agreement of only 60%. According to the interpretation of Cohen’s Kappa the observers show only a moderate concordance [24]. However, in the more intricate ten-segment system, agreement sharply declines, with only 16.1% of respondents reporting a matching segment combination. Similarly low values have been documented in the existing literature [13, 22, 25, 26].

Nevertheless, the literature suggests that both inter- and intrarater reliability of classification systems can be enhanced through three-dimensional fracture visualization of CT data sets. Techniques such as 3D printing and/or MR visualization have demonstrated the potential for further improvement [2, 3]. It is advisable to leverage the most advanced visualization technology available in routine clinical practice.

Notably, additional MRI imaging has been shown to augment classification reliability [27]. In this study, the specialists sought additional preoperative MRI imaging in 42.9% of cases. Additional meniscal and/or ligamentous injuries by TPF are identified as independent factors for early secondary total TKA [28]. The incidence of these injuries is reported to be as high as 90% [29–31] and postoperative instability can be detected in the majority of patients [32]. But still there is no current clear recommendation for preoperative MRI [1].

Remarkably, this study reveals that a high percentage (76.8%) of primary external fixators was recommended solely based on the fracture pattern. The participants were not given information on soft tissue conditions, peripheral blood circulation, motor function, or sensitivity.

Moreover, primary TKA was not chosen in any of the cases. This might be attributed, among other factors, to the absence of information on patient age. However, it also underscores that osteosynthetic treatment continues to be considered the standard procedure, e.g. in this kind of fracture types.

Focusing on the treatment strategy, all respondents aim for comprehensive 360° treatment; however, variations arise in its implementation. These differences start with the patient’s positioning, while the complexity deepens when considering the choice of approach and plate position. The most common combination is a posteromedial and anterolateral approach with plate position, yet surgeons may opt to perform this with or without repositioning. The anterolateral approach emerges as the most frequently chosen, aligning with findings in the literature for treating TPF [20, 33, 34].

Despite the recognition of the importance of addressing posterior fracture fragments for improved patient outcomes [16, 35], the majority of respondents combine the anterolateral approach with a posterior, usually posteromedial, approach. However, studies indicate limitations in the visibility of the tibial plateau through standardized approaches [36–38], such as approximately 36% visibility of the joint surface with the anterolateral approach [36]. Frosch proposed “the concept of direct approach to the fractures and stepwise extension as needed” [39], with lateral condylar osteotomy capable of achieving an additional joint space opening of 5–7 mm [40]. Interestingly, this study reveals that extended approaches are not intended in the majority of cases. The German S2k guideline recommends extending the approach if the fracture is difficult to assess, but it does not specify the preferred method of extension [41]. Consequently, a standard extension of the approach is not commonly performed, possibly due to the absence of recommendations regarding whether a condylar or fibular osteotomy should be preferred.

There is little agreement on the methods of intraoperative imaging, reaching a maximum of 40% for case III. In all three cases, the image intensifier is used most commonly, supplemented in two out of three cases by an intraoperative 3D scan. Studies indicate that unsatisfactory reduction results are missed when using image intensifier controls only [42, 43]. Improved anatomical reduction can be achieved with additional intraoperative fracturoscopy [42]. Nanoscopy appears promising for enhancing visibility in the posterior-lateral-central region, even though it may still face challenges [44]. Despite the highest radiation exposure, intraoperative CT scans are the most accurate method for detecting malreduction and malpositioning of implants [40]. In complex fractures, such as those presented in this paper, it may not be sufficient to control the reduction of the image intensifier alone. It remains unclear whether the infrequent use of fracturoscopy and/or 3D scanning was due to a lack of knowledge of the technique or the unavailability of equipment.

The treatment strategy appears to be influenced by the hospitals level of care. Level II and III hospitals are significantly less likely to request a preoperative MRI, possibly due to the limited MRI capacity in their infrastructure. Investigating other notable differences between care levels, such as positioning, surgical approach and control of reduction, emphasises the importance of respondents' professional experience. Interestingly, there is no significant difference in professional experience between hospitals in level I and care levels II + III, indicating that the variations cannot be attributed to the number of operations performed by the individual surgeon.

This study has several limitations. Firstly, the small number of fracture cases introduces a selection bias, and the limited number of participants further compounds this constraint. Additionally, no information on patient age, trauma history, or clinical examination was provided during the interviews. This makes it difficult to decide whether a plate osteosynthesis or an endoprosthetic therapy is the right choice. The inconsistency in fracture classification also contributes to the variation in treatment methods, as the classification serves as a foundation for following decisions. Besides, surgeons were not asked whether they have practical experience in using an extended approach. Therefore, the present study cannot clarify whether the low use of extended approaches is due to surgeons’ lack of surgical knowledge or to the inconclusive data on the use of these approaches. Moreover, the hospitals and surgeons were not randomly chosen. It would be valuable to explore differences among a larger and more diverse group of specialists, not just from a single region but also from various countries. 

Despite the limitations, the data from this study show that even among highly experienced surgeons, significant variations exist in the management of TPF. Although guidelines have been recently published [1], there is a lack of standardization concerning the overall management of TPF. As such, the authors of this paper advocate for the establishment of a national register to compile comprehensive and standardized data in the surgical management of TPF.

## Conclusion

Significant variations exist in management of TPF. While a comprehensive 360° treatment approach is universally implemented across specialists, there is a lack of standardization concerning preoperative imaging, classification, initial treatment, approaches, osteosynthesis, and reduction control. Subsequently, the current lack of standardization underscores the ongoing need for uniform data in the surgical treatment of TPF, aiming to improve and reducing complications.

## Electronic supplementary material

Below is the link to the electronic supplementary material.

Supplementary file1 (TIFF 1956 KB)

Supplementary file2 (TIFF 1956 KB)

Supplementary file3 (TIFF 1956 KB)

Supplementary file4 (TIFF 1956 KB)

## Data Availability

No datasets were generated or analysed during the current study.

## Appendix Table 4

**Table 4:** Overview of most frequently selected answer option

	Case 1 (n = 23)	Case 2 (n = 23)	Case 3 (n = 10)
Schatzker classification	52.2% (n = 12) as type 5	56.5% (n = 13) as type 5	60% (n = 6) as type 5
Ten-segment-classification	100% (n = 23) ALC, ALL, PLL	96% (n = 22) PC	100% (n = 10) PLC
Additional MRI	30.4% (n = 7)	56.5% (n = 13)	40% (n = 4)
Primary care	78.3% (n = 18) external fixator	82.6% (n = 19) external fixator	60% (n = 6) external fixator
Positioning	43.5% (n = 10) prone position to supine position	52.2% (n = 12) supine position	70% (n = 7) prone position to supine position
Approach	69.6% (n = 16) dual approach 39.1% (n = 9) anterolat. + posteromed	82.6% (n = 19) dual approach 39.1% (n = 9) anterolat. + posteromed	70% (n = 7) dual approach 50% (n = 5) anterolat. + posteromed
Extended approach	65.2% (n = 15) none	91.3% (n = 21) none	60% (n = 6) none
Osteosynthesis	65.2% (n = 15) double plate	87% (n = 20) double plate	60% (n = 6) double plate
Additional screw osteosynthesis	69.6% (n = 16)	60.9% (n = 14)	60% (n = 6)
Bone augmentation	69.6% (n = 16) allogeneic	47.8% (n = 11) allogeneic	70% (n = 7) allogeneic
Intraoperative imaging	34.8% (n = 8) image intensifier; image intensifier + 3D	39.1% (n = 9) image intensifier	40% (n = 4) image intensifier + 3D

*ALC* Anterior-lateral-central, *ALL* Anterior-lateral-lateral, *PLL* Posterior-lateral-lateral, *PC* Posterior-central, *PLC* Posterior-lateral-central
