# Supplementary figures and images for: Lack of standardisation in the management of complex tibial plateau fractures: a multicentre experience
Source: Eur J Trauma Emerg Surg. 2024 Aug 2;50(6):2937–45. doi: 10.1007/s00068-024-02616-6 (PMC11666675; doi:10.1007/s00068-024-02616-6)

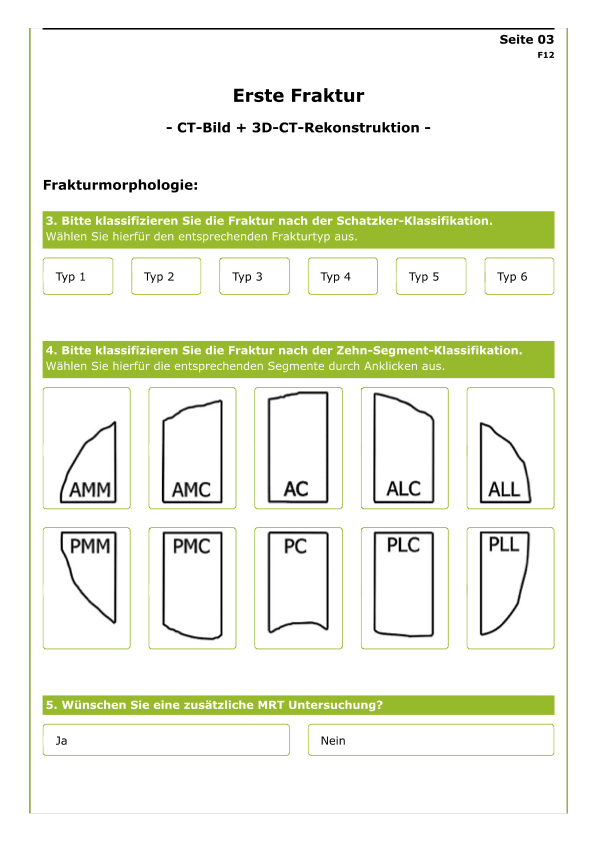

Supplement: Supplementary file 1 — Supplementary file1 (TIFF 1956 KB) [file 68_2024_2616_MOESM1_ESM.tiff]

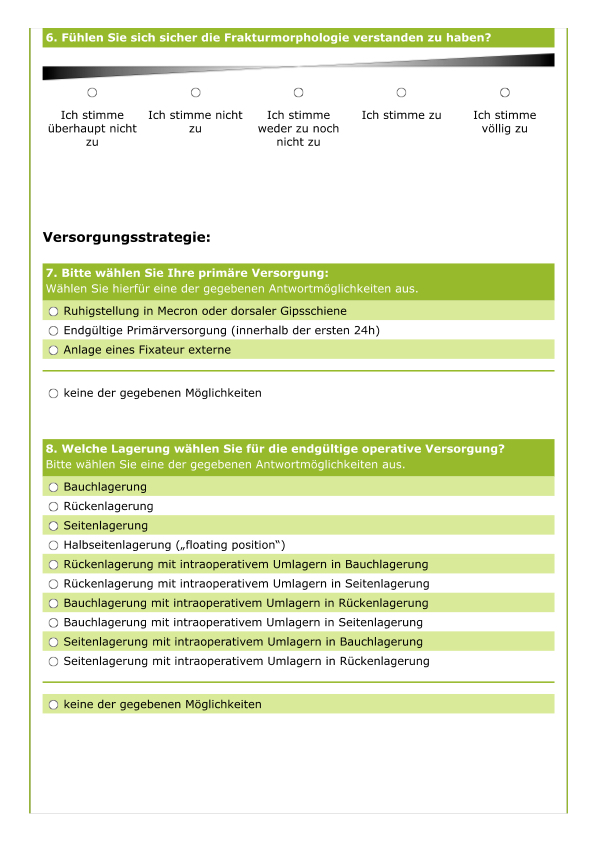

Supplement: Supplementary file 2 — Supplementary file2 (TIFF 1956 KB) [file 68_2024_2616_MOESM2_ESM.tiff]

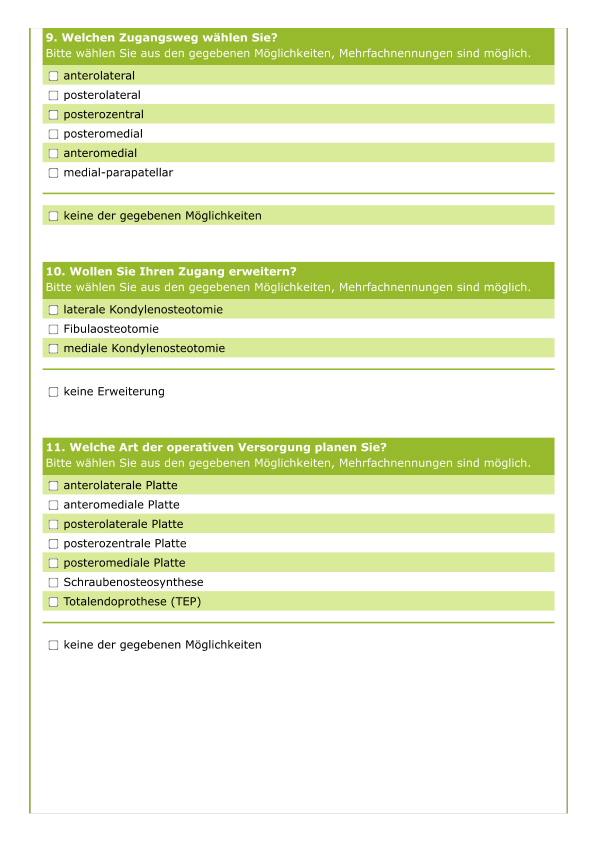

Supplement: Supplementary file 3 — Supplementary file3 (TIFF 1956 KB) [file 68_2024_2616_MOESM3_ESM.tiff]

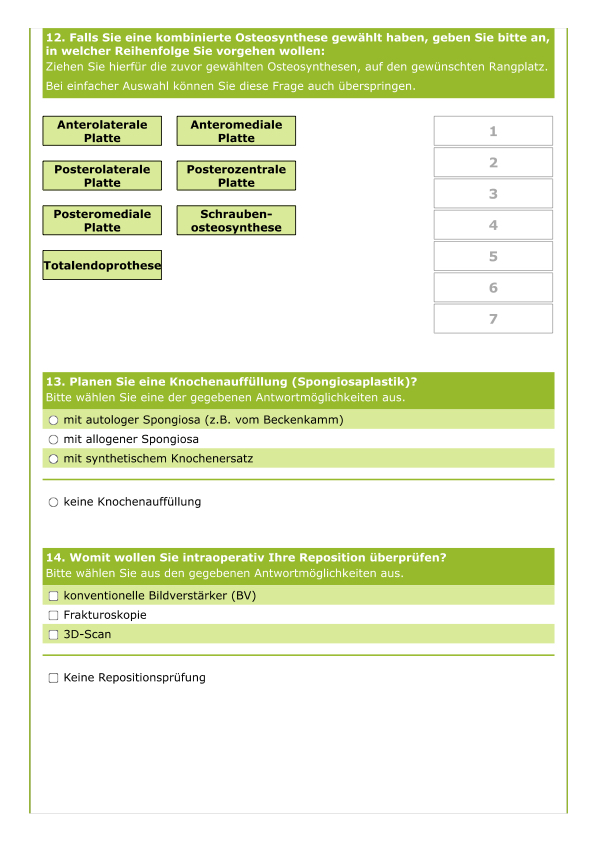

Supplement: Supplementary file 4 — Supplementary file4 (TIFF 1956 KB) [file 68_2024_2616_MOESM4_ESM.tiff]
